# Physics of the mechanical toy Gee-Haw Whammy Diddle

**DOI:** 10.1038/s41598-018-22079-1

**Published:** 2018-02-27

**Authors:** Martin Marek, Matej Badin, Martin Plesch

**Affiliations:** 1 Joint School Novohradská, GJH, Bratislava, 821 09 Slovakia; 2 Comenius University, Faculty of Mathematics, Physics and Informatics, Bratislava, 842 48 Slovakia; 3 Slovak Academy of Sciences, Institute of Physics, Bratislava, 845 11 Slovakia; 4 Masaryk University, Institute of Computer Science, 602 00 Brno, Czech Republic

## Abstract

Gee-Haw Whammy Diddle is a seemingly simple mechanical toy consisting of a wooden stick and a second stick that is made up of a series of notches with a propeller at its end. When the wooden stick is pulled over the notches, the propeller starts to rotate. Despite its simplicity, physical principles governing the motion of the stick and the propeller are rather complicated and interesting. Here we provide a thorough analysis of the system and parameters influencing the motion. We show that contrary to the results published on this topic so far, neither elliptic motion of the stick nor frequency synchronization is needed for starting a stable motion of the propeller.

## Introduction

Scientific toys play a very important role in science education and popularization. They are often used for demonstration purposes connected with a specific scientific phenomenon or principle. Students or general audiences can get a hands-on feeling of eddy currents by throwing a magnet into a metallic tube and observing its slow motion, or a better understanding of magnetic fields by levitating a magnetic spinner.

**Figure 1:**
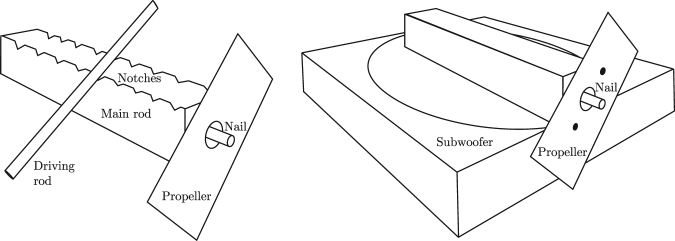
On the left, a typical gee-haw whammy diddle; on the right, a rod attached directly to a subwoofer.

Gee-haw whammy diddle is a scientific toy of a slightly different type. It consists of a wooden base-stick equipped with a series of notches on one of its sides with a propeller loosely attached to its end so that it can freely rotate (Fig. 1). Another stick is used for rubbing over the notches, which causes the propeller to rotate. Skilled performers are able to achieve very high rotation speeds of the propeller and change the rotation direction very quickly.

Being a mechanical device, one would expect to be able to pinpoint a simple and clear explanation of why and how linear motion along the notches induces a circular motion of the propeller and what defines the direction of this motion. This is however not the case despite research on this topic lasting almost a century. In one of the first works on this topic, Leonard^1^ suggests that the propeller vibrates due to an elliptical motion of the end of the stick and the direction of the motion is determined by the phase shift between the vertical and horizontal stick vibrations, caused by different rubbing directions.

This idea was further developed by Schlichting *et al*.^2^ where the direction of rotation is suggested to be related to the way the stick is being held by hand. Authors also provide a basic numerical output given the possibilities of computers of that period. Almost a decade later two other papers appeared on this topic. Jun Satonobu *et al*.^3^ provided presumably the most complex analysis of the problem so far, from both a theoretical and an experimental point of view. They confirmed the validity of the original hypothesis – that the rotation direction is determined by the elliptical movement of the end of the stick and the speed of the propeller by the magnitude of the areal velocity of the end of the stick. However, the presented experiment concluded only a one-sided implication of the hypothesis, namely that an elliptical movement of the stick causes a rotation of the propeller in the given direction, but did not show that this is the primary cause of the movement in the real toy.

Further analytical and experimental research was performed by Wilson^4^, whose results partially contradict the previous work in claiming that the direction of rotation of the end of the stick does not necessarily define the direction of the propeller’s motion. He also presented the hypothesis that the frequency of rotation of the propeller is synchronized with the driving frequency of the notches. However, this work has a few limiting factors embedded. In the analytical part, no slip is assumed between the stick and the propeller, which makes the problem and its solution similar to the hula-hoop problem; however, there is no experimental evidence for this assumption. In the experiment, recordings were performed with very low temporal resolution (30 fps), which apparently led to rather imprecise results of the motion of the stick. Recently in the work by Bhattacharjee^5^, the problem was reconsidered by connecting it to the Kapitza pendulum and recalling the idea of synchronization between the motion of the stick and the propeller.

In this paper, we perform full experimental and numerical analysis of the gee-haw whammy diddle. We concentrate on the way how different types of movement of the end of the stick cause the propeller to rotate and analyze not only the rotation direction but also the angular speed depending on various parameters of the movement (amplitude, frequency, areal velocity, etc.). We show that most of the hypotheses presented in the literature so far fail under rigorous experimental tests – areal velocity (elliptical movement) of the stick is not needed to excite the propeller to high-speed rotations, and the rotation speed is not related to the frequency of vibrations. We suggest that the rotation is a result of a simple positive-feedback mechanism between the motion of the stick and the propeller and that its direction (in the absence of an areal velocity) is given by random fluctuations. The speed of rotation is determined by a combination of amplitude and frequency of the stick vibration, and the highest speeds are achieved when the typical stick acceleration is approximately equal to the gravitational acceleration.

## Results

Gee-haw whammy diddle in its original form is practically an uncontrollable device. The fact that it is held in one hand and rubbed by the other does not allow any reasonable control over the excitation parameters – neither the exact position of the stick nor its movement. In previous works^3,4^, different mechanical devices were suggested that partially fixed the issue of holding the stick by hand, but kept the rather complicated way of stick excitation by rubbing. Although this does mimic the original idea of the device, it is connected to many complications. First, as the rubbing stick needs to be moved in a periodic movement, the frequency of the oscillations (caused by running over the notches) is far from being constant. The amplitude of the oscillations also depends on the position of the stick (notches closer to the end of the stick cause a smaller amplitude than the ones closer the fixing point) and on its speed (higher speed means stronger collisions). By keeping this system in its original form, it is nearly impossible to separate the relevant parameters that influence the motion of the propeller.

Therefore, we decided to redesign the experiment to concentrate solely on the motion of the end of the stick. Instead of having it rubbed over its notches, we attached the stick to a subwoofer and vibrated it in different frequencies and amplitudes (Fig. 1).

We also changed the position of the stick on the subwoofer (from the center towards the end) to simulate different combinations of horizontal and vertical vibrations. The motion was recorded by a high-speed camera that allowed the tracking of both the end of the stick and the propeller.

This configuration has several advantages. First, both the amplitude and the frequency of the oscillations are well controllable. The frequency is uniquely defined by the frequency of the sound from the signal generator; the amplitude of the oscillations depends on more parameters – amplitude of the signal, its frequency and exact positioning of the device on the subwoofer – but is perfectly stable once these parameters are fixed. Importantly, the movement of the stick is harmonic in both directions, contrary to the intrinsically inharmonic movement resulting from collisions between the sticks. These factors allowed us to perform well-controlled experiments and to distill the leading physical effects that influence the motion of the propeller.

**Figure 2:**
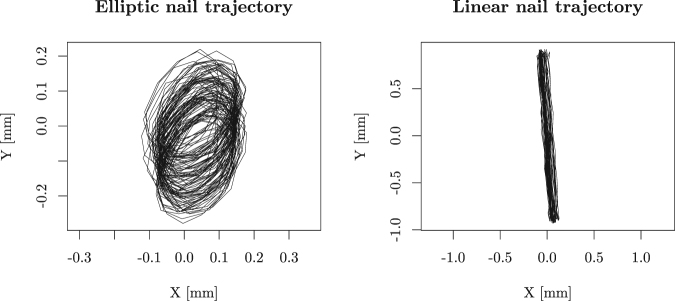
On the left-hand side, the position of the nail is depicted for the case where the stick was placed on the side of the subwoofer. Here we can see that the movement has an elliptical character with a well-defined areal velocity. On the right-hand side, movement of the nail is depicted when the stick was placed in the center of the subwoofer, where the areal velocity is close to zero.

We start the presentation of our results by general observations on the movement of the propeller depending on the relevant parameters. In Fig. (2), the readings of the position of the nail are presented for two cases – one is elliptical, resulting from positioning the stick on the side of the subwoofer, and one straight and nearly vertical, when the stick is placed in the center. This shows that with our experimental device, we were able to mimic the outcomes produced in the close-to-original gee-haw whammy diddle in refs^3,4^. For elliptical movement, we confirmed the results obtained so far – the direction of the rotation of the propeller was predefined by the direction of the elliptical movement (areal velocity). However, very interestingly, we were able to observe significant speeds of the propeller also in the case where no areal velocity was present in the movement of the nail (right-hand side of Fig. 2). In this case, the direction of the movement was random when starting from a still position and did not change if an initial push was given to the propeller. Therefore, we concluded the following proposition:

### Proposition 1.

*Elliptical movement of the nail is not necessary for starting and maintaining the rotation of the propeller. The speed of the propeller is not necessarily related to the areal velocity of the movement of the nail*.

**Figure 3:**
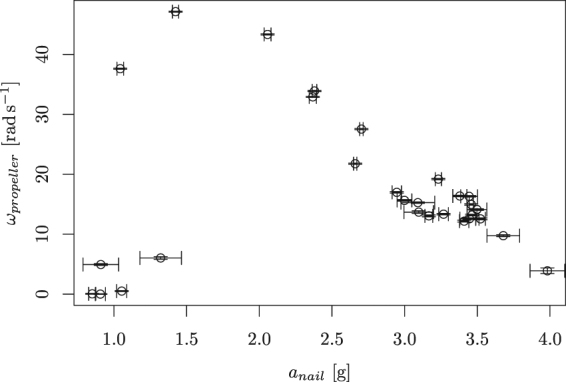
Dependence of the propeller speed on the maximal acceleration of the nail (measured in factors of the gravity constant *g*) during its movement. The propeller will not rotate if this acceleration is smaller than one, i.e., if the propeller would not de-touch from the nail even while going down. By further increasing the acceleration, the speed of the propeller is decreasing.

In what follows we concentrated our effort on a purely linear movement of the nail, opening a very interesting fundamental question – what is the principle that allows such a simple movement to cause the propeller to rotate? The sole two remaining parameters of the nail movement are its frequency and amplitude. We performed an extensive set of measurements with different combinations of these two parameters and were measuring the absolute value of the angular velocity of the propeller. We introduced a joint parameter of the nail movement, namely its maximal acceleration

1 $${a}_{n}=4{\pi }^{2}{A}_{n}\,{f}_{n}^{2},$$

where *A*_*n*_ is the amplitude of the nail’s movement and *f*_*n*_ its frequency. All results obtained are combined in Fig. 3. Based on these results we formulated the following proposition:

### Proposition 2.

*The propeller only rotates if the nail de-touches from the propeller during its movement, thus moves down with acceleration exceeding gravity. Further increase of the acceleration leads to smaller propeller speeds*.

This proposition can be explained by a model where the movement of the propeller is caused by collisions between the nail and the propeller, rather than by continuous movement of the nail along the propeller hole (similar to the way how hula-hoop is explained). In this model, the nail needs to de-touch from the propeller while moving down to allow an impact when returning back. This model also qualitatively explains why the optimal speed is reached for accelerations between 1 *g* and 2 *g*. First, it is important to notice that the mean absolute value of the acceleration is $\frac{1}{2}{a}_{n}$, so if the maximal acceleration is 2 *g*, the “typical one” is *g*. If the typical acceleration is around *g*, the time the propeller stays de-touched from the nail during its way down is maximized. For higher values of acceleration, the nail quickly reaches the bottom edge of the hole in the propeller, and the driving cannot proceed. Also, for higher acceleration, the forces during the “touching phase” are larger and cause a higher deceleration of the propeller due to friction.

**Figure 4:**
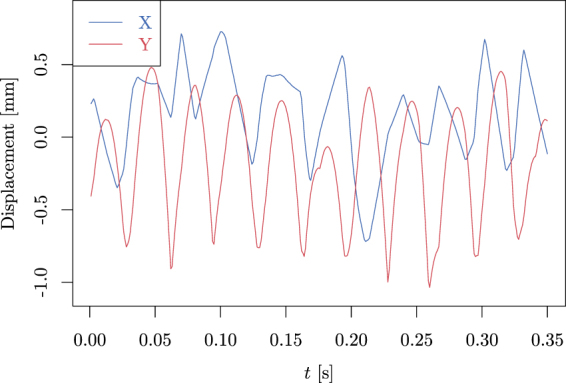
Here we display the position of the center of the propeller vs. time. It can be seen that the x-coordinate evolves closely to free motion, interrupted by collisions. In the y-coordinate, the movement resembles parabolas (free fall motion), again interrupted by collisions.

To examine this model in more detail, we focused on the movement of the propeller. As its tilt and rotational speed are biased by fluctuations explained above, we first concentrate on the translational movement. In Fig. 4, we present the motion of the center of the propeller vs. time. It can be seen that the motion during most of the time is governed by the free fall equation (constant speed in the x-direction and constant acceleration in the y-direction), interrupted by occasional collisions and short periods of continuous touching. Therefore, we formulate the proposition:

### Proposition 3.

*For larger speeds of the propeller, its motion can be described by free fall motion and short collisions with the nail*.

Based on the propositions described above we built a theoretical model. The model is based on the assumption that the movement of the propeller is driven mainly by short collisions between the propeller and the nail and most of the time moves freely. This assumption is backed up by the observation of the translational movement depicted in Fig. 4, but also on the direct observation of video recordings from which it is clear that most of the time, the propeller does not touch the nail.

Naturally, the crucial point in this model is to describe the collisions, as (up to air friction) the movement in between the collisions is trivial. Experimental data allows us to describe with high precision the position of the center of the nail and, knowing its diameter, also its boundary. We can also describe the center of the propeller and the boundary of the hole, but due to imperfections based on the readout delay, exactly in the frames in which a collision occurred, this description is inaccurate. So basically, for most of the frames, we get perfect data that allows us to pinpoint both the nail and the propeller, but for the interesting frames, including collisions, this is not the case.

Therefore, we had to build a model that does not rely on detecting and describing the collisions based on comparing the positions of the nail and the propeller, but rather on the abrupt change of translational velocity of the propeller. For a very short collision that happens at an angle *ϕ* between a vertically moving nail and the propeller, we model the impact by two forces: $\int F\,dt$ acting on the propeller outwards from its center and *f* $\int F\,dt$ in the perpendicular direction, where *f* is the friction coefficient between the nail and the propeller, which can be negative – the sign determines in which direction the friction force is acting. In general, the friction force can be smaller as well, but this only happens if there is no slipping on the touching point, which does not occur even for propellers moving at rather small angular velocities.

These two impacts will change both the momentum in the x- and y-direction of the propeller, as well as the angular momentum of the propeller in the following way:

2 $${\rm{\Delta }}{p}_{x}=\,\cos \,\varphi \int {F}\,{dt}+\,\sin \,\varphi \,{f}\int {F}\,{dt}$$

3 $${\rm{\Delta }}{p}_{y}=\,\sin \,\varphi \int {F}\,{dt}-\,\cos \,\varphi \,{f}\int {F}\,{dt}$$

4 $${\rm{\Delta }}L=rf\int F\,dt,$$

where *r* is the radius of the hole in the propeller. There is no experimental way to directly measure $\int F\,dt$ for an individual collision, but we can treat it as a variable and solve for it from the first two equations, and plug it into the last one. We get

5 $${\rm{\Delta }}L=r({\rm{\Delta }}{p}_{x}\,\sin \,\varphi -{\rm{\Delta }}{p}_{y}\,\cos \,\varphi ),$$

which can be directly calculated for each frame, knowing Δ*p*_*x*_, Δ*p*_*y*_ and *ϕ*. Change of momentum in the x-direction in the collision is simply given by its change between the frames, as there are no other forces acting in this direction. This is not the case for Δ*p*_*y*_, where we need to subtract the contribution of the gravitational acceleration given by

6 $${\rm{\Delta }}{p}_{y}^{g}=mg{t}_{f},$$

where *m* is the mass of the propeller and *t*_*f*_ = 1 ms is the time of one frame. The angle *ϕ* can also be determined from the position of the nail and the propeller. It is interesting to see that the resulting change in angular momentum (5) is independent of the friction coefficient *f*.

Let us recall here that the sum of the momenta in the x-direction (2), as well as in y-direction (3) corrected for the gravitational force (6) shall be close to zero for any subsequent set of frames, which can serve as a sanity check on the data. On the other hand, if the propeller accelerates its rotation or even keeps it constant, the sum of angular momentum contribution should be positive or negative (depending on the direction of the rotation) and increase in value for an increasing number of subsequent frames.

### Experimental Results

**Figure 5:**
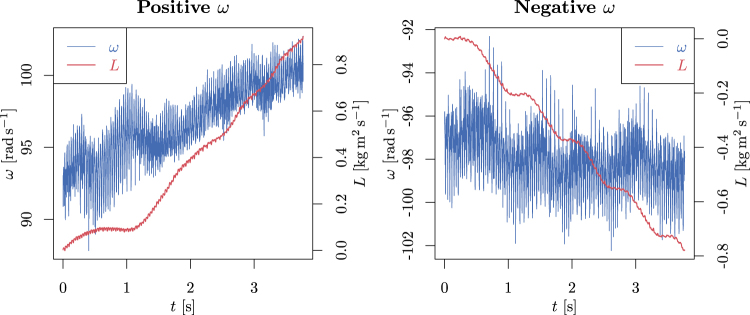
Here we depict the time dependence of the propeller rotation speed *ω* and the aggregated angular momentum Δ*L*. While the precision of the propeller frequency is not very high, the increasing magnitude of angular momentum is clearly visible. Recall that part of this momentum is continuously lost in friction. It can be seen that both the propeller speed and the aggregated angular momentum through equal time spans are basically the same for both rotation directions.

We have analyzed the angular momentum contributions (5) for different types of motion of the propeller – stable rotation in both directions and accelerating rotation from a still point. In Fig. 5, on the left, we can see that the aggregated angular momentum is clearly increasing with time for a positive-direction rotation. Part of this momentum is lost in friction and part is utilized for increasing the frequency of the propeller. The same kind of data is plotted on the right for a negative direction of propeller movement. Except for the direction, all other results are very similar: both the final frequency and the aggregated angular momentum per second.

**Figure 6:**
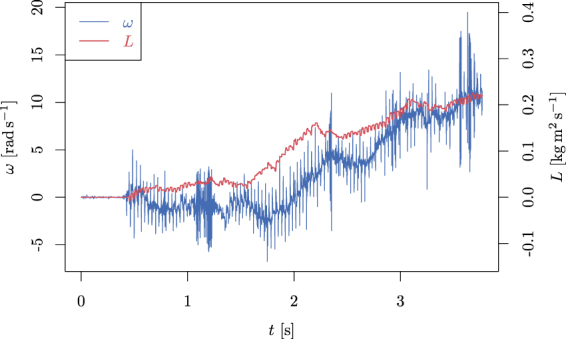
The starting phase of the propeller’s motion. It can be clearly seen that, especially in the first phase (first second), the aggregation of angular momentum is very small and the propeller does not move. However, also, during the next two seconds, the efficiency of the aggregation is smaller compared to the situation depicted in Fig. 5.

We have also analyzed the starting period of the movement in Fig. 6. Here less of the aggregated momentum is lost in friction (so the propeller accelerates), but on the other hand, the efficiency of the aggregation is much smaller, especially in the starting phase.

We further analyzed the experimental data from a different perspective. In principle, one can divide the recorded frames into two types: the ones with and the ones without collisions. In the former we expect ${\rm{\Delta }}{p}_{x}=\,0$ and ${\rm{\Delta }}{p}_{y}-{\rm{\Delta }}{p}_{y}^{g}=0$ due to equation (6), leading to ∆*L* = 0, whereas in the latter these values should be nonzero (and, for relevant collisions, not too small). However, taking the experimental errors into account, in all frames, both Δ*p*_*x*_ and ${\rm{\Delta }}{p}_{y}-{\rm{\Delta }}{p}_{y}^{g}$ are naturally non-zero. One would expect that the influence of these frames is negligible for the total aggregated angular momentum. To test this hypothesis, we can set a threshold for the total change in the momentum per frame and count the aggregated momentum only from the frames for which this threshold is reached. The other equivalent option is to take a specific number of frames with the highest change of translational momentum

7 $${\rm{\Delta }}{p}_{tot}=\sqrt{{\rm{\Delta }}{p}_{x}^{2}+{\rm{\Delta }}{p}_{y}^{2}}$$

and look at the total aggregated angular momentum (5).

**Figure 7:**
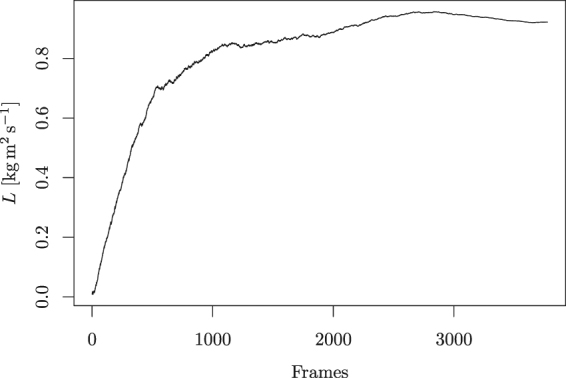
Here we show the aggregation of angular momentum for the positive direction of rotation based on the frames with the highest change of translational momentum, corresponding to the strongest collisions. One can see that most of the angular momentum is aggregated in about 10–15% of the frames with the highest change in translational momentum, which corresponds to the observation that in about this fraction of the frames a collision occurs.

In Fig. 7, we show the dependence of the total aggregated angular momentum on the number of frames taken into account. It can be clearly seen that the main aggregation happens in about 15% of the frames with the highest change of translational momentum, corresponding to the collisions. This also corresponds to the observations of the video recordings, showing that a collision happens a few times during one period of the nail movement. The contribution of the rest of the frames is composed of the contribution of weaker collisions and noise, and in total is insignificant. This confirms the proposition that the propeller gains most of its angular momentum during short collisions rather than by continuous touching of the nail.

### Simulation

To further support the hypotheses presented so far, we decided to create a numerical simulation of the propeller’s motion. We simulated the essence of our model – vertical oscillations of the nail – to see whether this basic scenario, without taking into account any other effects connected with friction or elasticity, can lead to significant rotation speeds of the propeller.

In the simulation, parameters of the nail and its motion and the propeller were set to be the same as in the experiment. The other two input parameters include the coefficient of static friction *f* between the nail and the propeller and the coefficient of restitution *c*_R_, describing the level of (in) elasticity of the collisions. For determining the coefficient of friction, we used the existing experimental data, as the value of this coefficient was solved for in Eqs (2–4). The values were rather volatile for each frame, but taking the figures from 10% of the frames with the highest change of translational momentum resulted in a reasonably stable value of around *f* = 0.8.

**Figure 8:**
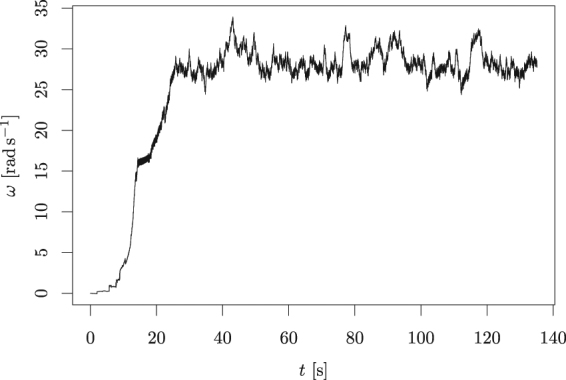
Start of the rotation of the propeller obtained by the simulation. We see that once the rotation starts, it reaches the value of 10 rads^−1^ within a few seconds, similarly to the experiment in Fig. 6. The terminal velocity, however, is smaller than the one reached experimentally.

Determination of the Coefficient of restitution is a bit more complicated, as this parameter was not directly used in the calculations. It is also experimentally very unstable because it effectively also includes the elasticity of the nail (part of the energy of the collision is temporarily stored in bending of the nail). Therefore, we decided to run the simulation for different (physically reasonable) values of this coefficient and concluded that the most reasonable results are obtained for *c*_R_ = 0.8. In Fig. 8 we show the result for the initial condition is (*x*, *y*) = (−0.001*R*, 0) and (*v*_*x*_, *v*_*y*_) = 0. The minor non-zero value in the starting point is necessary to introduce some asymmetry into the system, which is generated by random fluctuations in the real experiments.

The primary output of the simulation is the basic proof of the concept that a simple, symmetric motion of the nail with a perfectly zero angular velocity can result in rotational motion of the propeller. Detailed quantitative comparison of the results of the simulation and the experiment is difficult due to the fact that the simulation does not take into account other external influences like air friction, energy dependence of coefficient of restitution, elasticity of the nail, etc. In spite of this fact, we see by comparing Figs 6 and 8 that, once the asymmetry reaches some threshold, an angular speed of around 10 rads^−1^ is reached within a few seconds. However, the simulation saturates around 30 rads^−1^, whereas the experiment reached more than 100 rads^−1^.

We also performed the simulation using different combinations of *f* and *c*_R_, achieving some valuable insight into the system. Expectably, the bigger the coefficient of restitution, the more easily the rotation of the propeller is induced. However, the fluctuations around the average value of ω increase as well, making it very hard to even speak about a stable rotation. On the other hand, if the coefficient of restitution is made lower, the rotation of the propeller cannot be induced, basically due to the fact that the propeller “sticks” on the nail. By changing the friction, we essentially obtained the same dependence, but due to a different effect. Lower friction makes the nail slip more easily on the propeller, and thus less angular momentum is induced by the same strength of collisions. For a very (unphysically) high friction, the slipping stops, resulting in stopping of the rotation. Thus, we can conclude that both of the parameters have their optimal values leading to the best results in rotation and these values are close to those used in the experiment, up to the precision of their determination.

## Discussion

Gee-haw whammy diddle is a very interesting mechanical scientific toy that demonstrates the transition of linear driving into rotational movement. Within this paper, we have performed an extensive research of the device, aiming to pinpoint the essential phenomenon that causes the propeller to rotate. Contrary to the hypotheses presented in the previous works on this topic, in particular, works by Wilson^4^ and Satonobu^3^, we have shown that an elliptical movement of the stick is not necessary to induce a rotational movement of the propeller – on the contrary, a purely linear motion of the stick induces the highest speeds of the propeller. We have also shown that contrary to ideas presented so far^4,5^, there is no synchronization effect between the motion of the stick and the propeller – by purely harmonic motion of the stick, the propeller was able to accelerate from rest to high frequencies (however not reaching the driving frequency), and the stabilized speed of the propeller was strongly dependent on the amplitude of the oscillations.

We showed that the highest speeds of the propeller can be reached for parameters of the oscillations that secure the typical acceleration of the stick to be roughly equal to the gravitational acceleration. This is because, as concluded from the motion of the propeller and direct observation, best results are achieved for situations where most of the time the propeller is not touching the nail at the end of the stick. In this case, the propeller is being translationally stabilized by short collisions with the moving nail that provide a source of angular momentum. The direction of rotation is, for a linear stick movement, random. Gee-haw whammy diddle is often compared to the hula-hoop, also a well-known effect used for physics demonstration. We believe that this comparison is rather imprecise because the governing physical effects are distinctly different in those two cases. One could probably compare the gee-haw whammy diddle better to the Devil stick juggling, where one stick is being held in hand and used to rotate another stick freely flying in the air.

## Methods

### Experiment

To keep the experimental setup as close to the original problem as possible, we analyzed the gee-haw whammy diddle frequencies. While rubbing the stick by hand, the hand usually makes a few (say up to three) full cycles (there and back) on the stick per second. There are usually in the order of 10 notches on the stick which results in approximately up to 60 impulses per second. This is however only a very rough estimate, as the hand does not move at a constant speed there and back (resulting in a continuous range of frequencies) and the inharmonic impulses themselves produce a broad Fourier spectrum of oscillations. Using a subwoofer with a frequency range of 20 Hz to 90 Hz allowed us to simulate the leading frequencies which turned out to be enough to reproduce the effect −30 Hz was chosen for experiments with frequency taken as a fixed parameter. This itself is an important observation, as some of the hypotheses^4,5^ suggested a synchronization effect between the oscillations of the stick and the rotation of the propeller, which would require a broad spectrum of frequencies (or their time dependence) for starting the rotation.

The amplitudes of the motion also mimic the original problem. While on the lower end there was no technical limitation, the maximal amplitudes were restricted by the intrinsic limits of the subwoofer. For a central placement of the stick and optimal frequencies of the subwoofer, the vertical amplitude reached as much as 10 mm, whereas, for high frequencies and border placement, it was closer to just a few mm. This again turned out to be sufficient as we were able to reach the optimal conditions for the propeller rotation well within the range.

**Figure 9:**
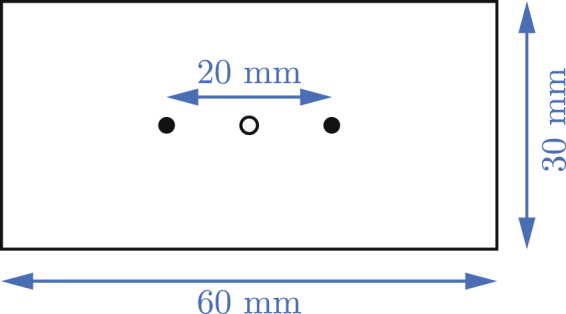
A 60 mm × 30 mm propeller with a 2 mm diameter hole.

It turned out that while the construction of the rod (a simple stick with a nail on its end) does not crucially influence the movement, this is not the case for the propeller (much neglected in the previous work). This is mainly due to the fact that if the center of gravity does not coincide with the center of the hole in the propeller, the propeller has a preferred hanging position on the notch, and the start of the rotation is more complicated. Even more importantly for small holes, if the center of gravity is outside of the hole, other mechanisms than those described above are needed to start the oscillations. As a result, we decided to use laser-cut propellers in the shape of a rectangle with dimensions of 60 mm × 30 mm × 2 mm and a hole of different diameters (2–10 mm) cut in the center with great precision, with weights of around 4.1 ± 0.1 g, depending on the diameter of the hole. Two points were also engraved on the propeller 20 mm apart (see Fig. 9) to allow tracking of the exact position of the propeller.

The whole motion was recorded on a camera with an optically stabilized lens system in 1000 fps and 1920 × 1080 resolution. The position of the nail and the points on the propeller were tracked with a tracking software (Tracker). This allowed us to track more than 10 points per period even for the highest oscillation speeds (and more than 33 for the most extensively used frequency) with precision in the order of a few μm. This is below the resolution of a single pixel (37 μm per pixel) since the tracking software analyzed the whole area of pixels and was able to determine the center of that area with a higher accuracy. All of this has led to a very precise description of the movement of the nail.

### Data Processing

Two points on the side of the propeller were used to determine two parameters – the position of the center of the propeller and its angle (determining the angular velocity, the final output measure). Three complications were present with the data processing.

First, the two engraved points were designed to be symmetrically displaced from the center of the propeller. Because a different device performed the engraving than the cutting, imperfections in the order of 0.5 mm arose. This was however easily corrected by analyzing a set of frames in the tracking software and adding a suitable offset to the position in the middle of these two points.

The second problem was related to the finite readout time of the camera. Readout of the whole image took about 600 ns, making it roughly t_*dr*_ = 600 ns per row. This means that in the vertical position of the propeller, the upper point was read out about 200 μs before the nail and the lower point was delayed by about the same time. This naturally biases both the position of the center of the propeller and its angle. This bias can be removed recurrently in the following way:

The tilt of the propeller *ϕ*^0^ is calculated for each frame from raw data without any corrections. From this data, the speed of the propeller ω^0^ can be determined in the 0th order. The time margin (or delay) of the point on the propeller can be calculated by

8 $${t}_{d}^{0}=({y}_{d}^{0}-{y}_{n}^{0}){t}_{dr},$$

where ${y}_{d}^{0}$ and ${y}_{n}^{0}$ are the raw positions of the dot and the nail (in pixels) respectively. Using this time delay and the estimate of the speed of the propeller ω^0^, one can calculate the first correction of the point on the propeller that it is expected to reach in the time when the point of the nail was recorded

9 $${x}_{d}^{1}={x}_{d}^{0}-R{\omega }^{0}{t}_{d}^{0}\,\sin \,{\varphi }^{0}$$

10 $${y}_{d}^{1}={y}_{d}^{0}+R{\omega }^{0}{t}_{d}^{0}\,\cos \,{\varphi }^{0},$$

where *R* is the distance between the point and the nail, which can be well approximated as the radius of the propeller (taken as half of the distance between the two engraved points). These two points can serve as the input for the next round of the recursive correction by obtaining a more precise tilt and speed of the propeller. However, it turned out that the first level is sufficient to correct the influence of this effect below other experimental imperfections.

There is also another problem connected with the processing of the tilt of the propeller. In certain cases, a collision happens between the readouts of the first and the second point on the propeller. Then the tilt in that specific frame is wrongly calculated, which causes jumps in the calculated speed of the propeller. There are two possible ways to tackle this problem. One, obvious, is to simply ignore the readouts where the speed of the propeller significantly deviates in the two subsequent points. The other is to detect the times of the collisions from the changes in the translational movement of the center of the propeller and ignore the readouts for those frames. Both, however, change the data acquired by discarding part of them. Despite using such procedures, we decided to present the data without any of these corrections, leading to more fluctuating, but fully transparent results.

### Simulation

In the simulation, we assume that a circular nail of radius *r*_*n*_ moves along a straight vertical line.

11 $$\begin{array}{rcl}{x}_{n} & = & 0\\ {y}_{n} & = & {A}_{n}\,\sin (2\pi {f}_{n}t+\alpha ).\end{array}$$

The propeller is a rectangle of dimensions *a* × *b* with a hole of a diameter *r*_*m*_ in its center and mass *m*, with a corresponding moment of inertia

12 $$I=\frac{1}{12}m\frac{ab}{ab-\pi {r}_{n}^{2}}({a}^{2}+{b}^{2})-\frac{1}{2}m\frac{\pi {r}_{n}^{2}}{ab-\pi {r}_{n}^{2}}\pi {r}_{n}^{2},$$

and a circular hole of radius $r > {r}_{n}$ positioned such that it coincides with the propeller’s centre of mass. Moreover, we denote the coefficient of static friction between the nail and the propeller as *f* and the coefficient of restitution (COR) between the nail and the propeller as *c*_*R*_.

The motion of the propeller can be divided into two phases that alternate: free fall of the propeller and short collisions.

#### Free motion

Since the propeller’s rotation can only be induced during collisions, the interesting part of the free motion is only the time between two successive collisions Δ*t*. This time is determined numerically, knowing the x- and y-position and the respective velocities *v*_*x*_ and *v*_*y*_ of the propeller together with the nail phase *α* at the time of the very last collision, by finding the smallest positive root of the following equation:

13 $${(x+{v}_{x}{\rm{\Delta }}t)}^{2}+{(y+{v}_{y}{\rm{\Delta }}t-\frac{1}{2}g{({\rm{\Delta }}t)}^{2}-{A}_{n}\sin (2\pi {f}_{n}{\rm{\Delta }}t+\alpha ))}^{2}-{(r-{r}_{n})}^{2}=0\,.$$

In more detail, using a finite step of 100 μs, the interval on which the left-hand side of the previous equation changes sign is determined. After finding the time interval, Δ*t* is determined with a relative precision of 10^−8^ using a binary search method. The position and velocity of the propeller prior to the very next collision are simply found as:

14 $$\begin{array}{rcl}x^{\prime}  & = & x+{v}_{x}{\rm{\Delta }}t\\ y^{\prime}  & = & y+{v}_{y}{\rm{\Delta }}t-\frac{1}{2}g{({\rm{\Delta }}t)}^{2}\\ {v^{\prime} }_{x} & = & {v}_{x}\\ {v^{\prime} }_{y} & = & {v}_{y}-g{\rm{\Delta }}t\\ \alpha ^{\prime}  & = & \alpha +2\pi {f}_{n}{\rm{\Delta }}t.\end{array}$$

#### Collisions

We assume that the collisions are short compared to the typical timescale of Δ*t*, therefore, the nail phase *α* does not change during a collision. In order to find the respective velocities ${v^{\prime\prime} }_{x}$ and ${v^{\prime\prime} }_{y}$ just after a collision, we move to a reference frame connected with the nail. The change of the reference frame only affects the y-component of the propeller velocity, ${v^{\prime} }_{y}\to {v^{\prime} }_{y}-2\pi {f}_{n}{A}_{n}\,\cos (\alpha ^{\prime} )$. For the sake of simplicity, we rotate the reference frame by the angle *ϕ*, defined by the touching point between the hole and the nail.

15 $$\begin{array}{rcl}{v}_{\perp } & = & {v}_{x}\,\cos \,\varphi +{v}_{y}\,\sin \,\varphi \\ {v}_{||} & = & -{v}_{x}\,\sin \,\varphi +{v}_{y}\,\cos \,\varphi .\end{array}$$

If we denote the rotation speed of the propeller *ω*, the tangential velocity of the touching point is ${v}_{\tan }={v}_{||}+\omega r$. In this reference frame, the equations (2–4) can be rewritten as

16 $${\rm{\Delta }}{p}_{\perp }=\int F\,dt$$

17 $${\rm{\Delta }}{p}_{||}=-{\rm{sgn}}({v}_{\tan })f\int {F}\,{dt}$$

18 $${\rm{\Delta }}L=-{\rm{sgn}}({v}_{\tan })rf\int {F}\,{dt}\,.$$

By introducing the coefficient of restitution, we implicitly model the impulse $\int F\,dt$ as $-m{v}_{\perp }\mathrm{(1}+{c}_{R})$. Knowing this fact and assuming that slipping occurs during the whole collision, one can solve for the velocities just right after the collision (in the reference frame of the nail) using the equations (16–18), obtaining

19 $${v^{\prime} }_{\perp }=-{c}_{{\rm{R}}}{v}_{\perp }$$

20 $${v^{\prime} }_{||}={v}_{||}-{\rm{sgn}}({v}_{\tan })f\mathrm{(1}+{c}_{{\rm{R}}})|{v}_{\perp }|$$

21 $$\omega ^{\prime} =\omega -{\rm{sgn}}({v}_{\tan })\frac{mr}{I}f\mathrm{(1}+{c}_{{\rm{R}}})|{v}_{\perp }\mathrm{|}.$$

In general, however, the slipping condition might not be fulfilled during the whole collision. This can be detected by a change of the sign of the tangential velocity (${\rm{sgn}}({v}_{||}+\omega r){\rm{sgn}}({v^{\prime} }_{||}+\omega ^{\prime} r\mathrm{)0}$), since the velocity cannot change its sign, only decrease its value to zero. If this is the case during the collision, one has an additional condition ${v^{\prime} }_{||}+\omega ^{\prime} r=0$, which brings a slightly different solution for the respective velocities just after the collision, namely

22 $${v^{\prime} }_{\perp }=-{c}_{{\rm{R}}}{v}_{\perp }$$

23 $${v}_{\parallel }^{\prime} ={v}_{\parallel }-{v}_{\tan }\frac{I}{I+m{r}^{2}}$$

24 $$\omega ^{\prime} =\omega -{v}_{\tan }\frac{mr}{I+m{r}^{2}}\,.$$

The numerical procedure then consists of rotating the reference frame by the angle −*ϕ*,

25 $$\begin{array}{rcl}{v^{\prime\prime} }_{x} & = & {v}_{\perp }^{\prime} \,\cos \,\varphi -{v}_{\parallel }^{\prime} \,\sin \,\varphi \\ {v^{\prime\prime} }_{y} & = & {v}_{\perp }^{\prime} \,\sin \,\varphi +{v}_{\parallel }^{\prime} \,\cos \,\varphi \end{array},$$

and moving back to the laboratory frame, ${v^{\prime\prime} }_{y}\to {v^{\prime\prime} }_{y}+2\pi {f}_{n}{A}_{n}\,\cos (\alpha ^{\prime} )$.

### Data availability

The datasets generated and analyzed during the current study are available from the corresponding author on reasonable request.
